# Development of an RNA Interference Tool, Characterization of Its Target, and an Ecological Test of Caste Differentiation in the Eusocial Wasp *Polistes*

**DOI:** 10.1371/journal.pone.0026641

**Published:** 2011-11-01

**Authors:** James H. Hunt, Navdeep S. Mutti, Heli Havukainen, Michael T. Henshaw, Gro V. Amdam

**Affiliations:** 1 Departments of Biology and Entomology and W. M. Keck Center for Behavioral Biology, North Carolina State University, Raleigh, North Carolina, United States of America; 2 School of Life Sciences, Arizona State University, Tempe, Arizona, United States of America; 3 Department of Chemistry, Biotechnology and Food Science, Norwegian University of Life Sciences, Aas, Norway; 4 Department of Biomedicine, University of Bergen, Bergen, Norway; 5 Department of Biology, Grand Valley State University, Allendale, Michigan, United States of America; University of Otago, New Zealand

## Abstract

Recent advancements in genomics provide new tools for evolutionary ecological research. The paper wasp genus *Polistes* is a model for social insect evolution and behavioral ecology. We developed RNA interference (RNAi)-mediated gene silencing to explore proposed connections between expression of hexameric storage proteins and worker vs. gyne (potential future foundress) castes in naturally-founded colonies of *P. metricus*. We extended four fragments of putative hexamerin-encoding *P. metricus* transcripts acquired from a previous study and fully sequenced a gene that encodes Hexamerin 2, one of two proposed hexameric storage proteins of *P. metricus*. MALDI-TOF/TOF, LC-MSMS, deglycosylation, and detection of phosphorylation assays showed that the two putative hexamerins diverge in peptide sequence and biochemistry. We targeted the *hexamerin 2* gene in 5^th^ (last)-instar larvae by feeding RNAi-inducing double-stranded *hexamerin 2* RNA directly to larvae in naturally-founded colonies in the field. Larval development and adult traits were not significantly altered in *hexamerin 2* knockdowns, but there were suggestive trends toward increased developmental time and less developed ovaries, which are gyne characteristics. By demonstrating how data acquisition from 454/Roche pyrosequencing can be combined with biochemical and proteomics assays and how RNAi can be deployed successfully in field experiments on *Polistes*, our results pave the way for functional genomic research that can contribute significantly to learning the interactions of environment, development, and the roles they play in paper wasp evolution and behavioral ecology.

## Introduction

Experimental studies conducted in natural environments are important for understanding ecological patterns and processes [1]. Experimental studies conducted on laboratory model organisms are important for understanding the selective basis of evolution [2]. *Polistes* paper wasps offer an opportunity to pursue a third realm of experimental studies now in its infancy – the implementation of genomics-based methods to experimentally study evolutionary mechanisms in natural environments.

**Figure 1:**
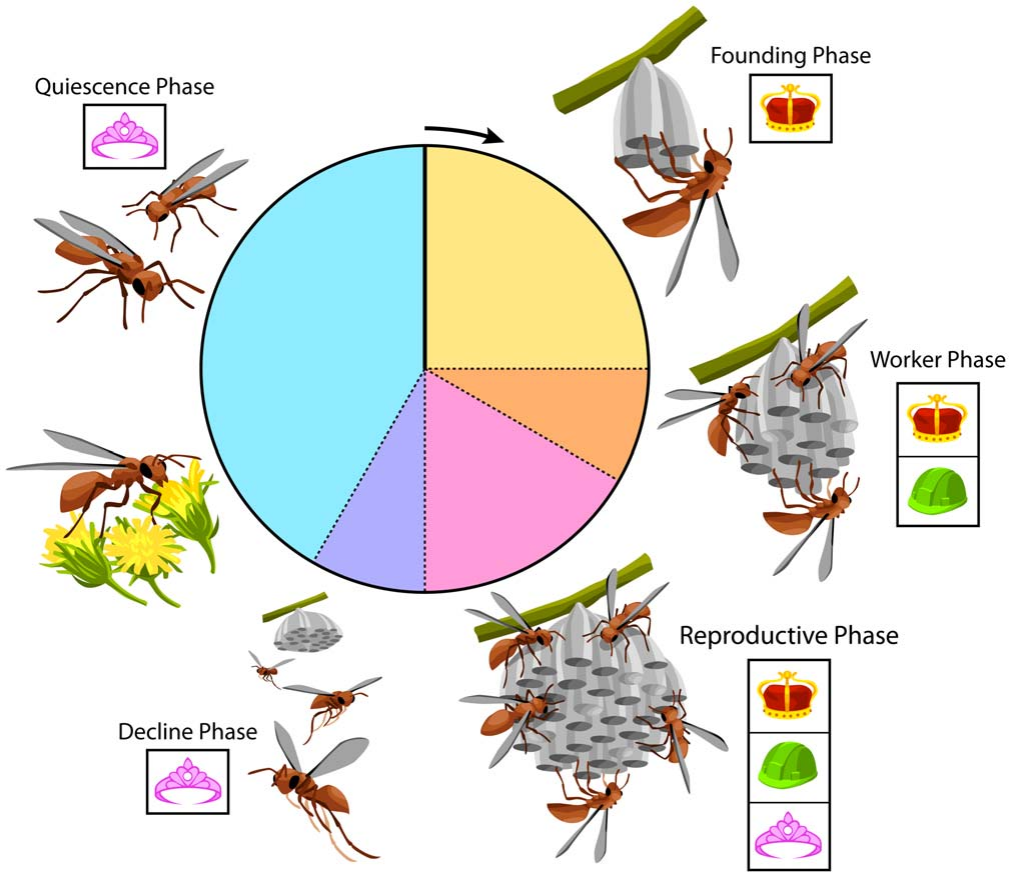
Colony cycle of an annual *Polistes* paper wasp, exemplified by *P. metricus*. Foundress Phase. The annual cycle begins (arrow) when a female emerges from quiescence with undeveloped ovaries. Feeding at flowers enables ovary development, which precedes nest founding. Oviposition occurs immediately upon initiation of each nest cell by the now-maternal foundress (crown icon) that performs all maternal behaviors in addition to oviposition, including foraging, feeding larvae, and nest construction. Worker Phase. The first female offspring to emerge in early summer are workers (hard hat icon) that forage, feed larvae, and construct the expanding nest. The foundress transitions into a true social queen that limits her activities to oviposition and feeding larvae using foods brought to the nest by workers. Reproductive Phase. Female offspring emerging in mid-summer are non-working future foundresses called gynes (tiara icon). Males, not shown, emerge synchronously with gynes. Decline Phase. In late summer the queen and workers die. Gynes and males depart the nest, which now is empty and unattended. Quiescence Phase. Gynes and males mate and feed at fall flowers. With the decline in flower availability and onset of cooler weather, males die, and gynes enter a torpor-like quiescence in sheltered concealment. Workers live only from their emergence until the end of the reproductive phase. A gyne from the reproductive phase of one year has the potential to become a foundress and then queen the following year, thus the tiara to crown transition can represent single individuals that pass from gyne to foundress to queen. Larval treatments and sampling reported in this experiment were conducted in the Reproductive Phase of the colony cycle.

The paper wasp genus *Polistes* has long been an important taxon for field studies of its ecology [3] and behavior [4], [5], [6]. It is an especially important taxon for understanding the evolution of insect sociality [7], [8] and as a model system in behavioral ecology [9], [10]. The paper wasp *Polistes metricus* Say, distributed in the Midwestern and Southeastern U. S., has for several decades been the specific focus of diverse studies on aspects of *Polistes* biology. Early work includes physiology [11], inclusive fitness [12], behavior [13], and sex ratio [14]. To extend and expand these and other research areas at both the species and genus levels, *P. metricus* was the first *Polistes* species subjected to analysis of its expressed sequence tags (ESTs – its expressed mRNA) [15], which then were annotated by reference to the genome of a related species, the honey bee *Apis mellifera* [16]. Using this EST resource, Toth et al. [15] documented differences in gene expression among the four categories of adult females that occur in an annual colony cycle of *P. metricus* (Fig. 1). Similarly, Hunt et al. [17] used the EST resource to document differences in gene expression between larvae destined to become workers and larvae destined to become gynes (pre-queens). These studies were ‘natural experiments’ that were based on collection of specimens from natural environments at particular points in the colony cycle, but they were not manipulative experiments designed to test causal connections.

The manipulative experimental approach of RNA interference (RNAi)-mediated gene targeting occurs post-transcriptionally and involves specific degradation of target mRNA. RNAi is triggered by introduction of double stranded RNA (dsRNA) and can be a near-universal method of gene silencing [18], [19]. Among social insects, RNAi has been reported for dsRNA feeding in larval honey bees *A. mellifera* [20], [21] and nymphs of the termite *Reticulitermes flavipes* [22], and by dsRNA microinjection in adult honey bees [23], [24], the red imported fire ant *Solenopsis invicta* Buren [25], and the termite *Cryptotermes secundus* [26]. The majority of these studies were initiated in a laboratory situation. To develop RNAi for use in natural settings could advance the research utility of RNAi for learning mechanisms that underlie important features of social insects' behavioral ecology.

*P. metricus* may provide an ideal opportunity for this initiative. ESTs for *P. metricus* are now available, and paper wasps' ecology with easily observed nests has made them the subject of extensive natural history studies [8] and a smaller number of experimental studies in captive rearing (e.g. [27]), laboratory settings (e.g. [28]), and natural environments (e.g. [29], [30]). Adults feed nectar directly to larvae [8], and this can be mimicked by feeding nectar-like liquids to larvae, providing a vehicle for dsRNA. Specific larvae can be tracked through time by means of ‘nest maps’, enabling individual marking of newly-emerged adults. These adults can then be tracked on their nests in field situations or collected for subsequent analyses.

Here, we develop RNAi for *P. metricus* for experimental applications in natural environments. This approach enables testing of causal relationships between candidate genes (genotype) and their association with behavior and/or caste ecology (phenotype). We exemplify this potential by exploring a central developmental question with significance in behavioral ecology: the role of specific storage proteins in developmental bias toward workers and gynes (future queens of the next generation). Storage proteins are ecologically important gene products that play a role in diapause not only in *Polistes* [31] but in a broad range of insects [32], [33]. *Polistes metricus* has two putative hexameric storage proteins, Hexamerin 1 and Hexamerin 2. Of these, Hexamerin 1 levels in late-larval and early-pupal stages provide a significant predictor of caste bias in adults, whereas Hexamerin 2 does not [31], leaving the roles(s) of the latter protein obscure. Fragments of putatively Hexamerin-encoding transcripts are available as ESTs, but it has been unclear how these relate to the two largely uncharacterized *Polistes* proteins.

We report successful cloning of the Hexamerin 2 encoding gene of *P. metricus* from its available ESTs, and proteomic analyses of the *P. metricus* Hexamerin 1 and Hexamerin 2 proteins. The proteins were identified as separate products with largely diverging peptide sequence and different biochemical properties. Subsequently, dsRNA was synthesized for *hexamerin* 2 and a control gene (*gfp – green fluorescent protein*), and these were used in a larval feeding experiment with naturally-founded *P. metricus* colonies in the field. We show that larval Hexamerin 2 can be successfully suppressed by RNAi and that the adult caste phenotype of Hexamerin 2 knockdowns is not significantly altered. These results as well as suggestive trends in our data support the proposition of diverging roles of the Hexamerin 1 and Hexamerin 2 products in caste development.

## Results

### Sequence of *hexamerin 2* transcript and protein

**Figure 2:**
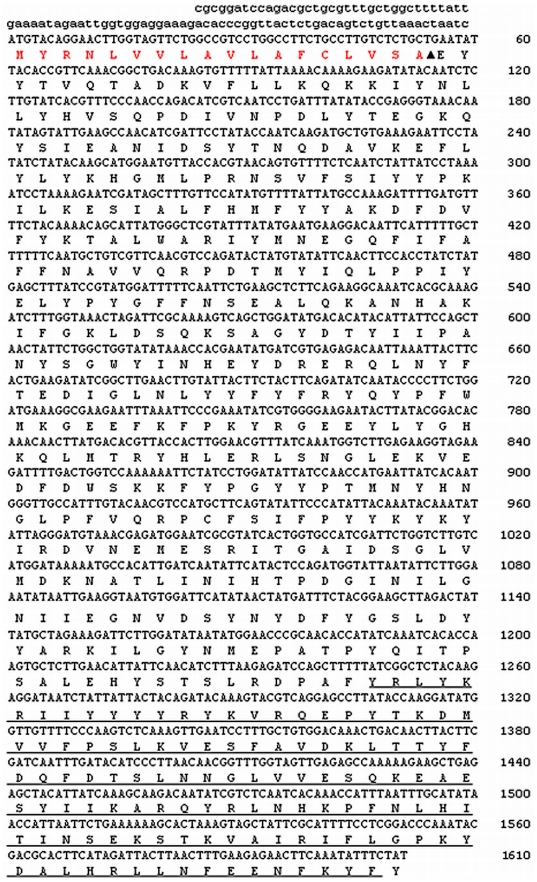
Nucleotide sequence of *hexamerin* 2 cDNA clone from *P. metricus*, along with the deduced protein sequence. The arrowhead indicates the signal cleavage site as predicted by SignalP (www.cbs.dtu.dk/services/SignalP). The underlined portion of the amino acid sequence corresponds to the dsRNA used for down-regulating *hexamerin 2*. The region corresponding to the 5′ UTR (Untranslated Region) is shown with lowercase letters. The *hexamerin 2* sequence shown is partial and does not include the C-terminal end (∼50 amino acids). The GenBank accession number for the *hexamerin 2* sequence is HQ661804.

The *P. metricus* EST database contains four partial sequences, Contig45616, Contig45913, Contig42334, and Contig40833, that share homology with *A. mellifera* hexamerins. We used RACE to complete the cDNA ends, resulting in a single cDNA (Fig. 2) that overlaps all four contigs. The N-terminal sequence of the encoded protein constitutes a putative secretion signal peptide for an extracellular protein (www.cbs.dtu.dk/services/SignalP/), with cleavage predicted between residues 18 and 19 (amino acids VSA-EY, Fig. 2). The predicted mass of the mature protein is ∼68.5 kDa, which is slightly lower than observed on SDS-PAGE gel. This apparent difference in size could be due to post-translational modifications attached to the protein sequence. Accordingly, we tested for N- and O-glycosylation and found potential N-glycosylation sites at asparagine residues at positions 201 and 344 (www.cbs.dtu.dk/services/NetNGlyc/).

The cloned gene belongs to the hemocyanin family and has three conserved domains: hemocyanin_N (an all-alpha domain; residues 29–151), hemocyanin_M (which binds to copper and is necessary for the oxygen transport in the hemolymph; residues 158–415), and hemocyanin_C (an ig-like domain; residues 435–519) [34]. The gene shows highest identity to *A. mellifera* hexamerin 70A (64% identity), while the degree of shared identity to other hexamerins is 55% to *Nasonia vitripennis*, 47% to *Camponotus festinatus*, and 44% to three mosquito sequences: *Aedes aegypti*, *Culex quinquefasciatus*, and *Anopheles gambiae* (Fig. S1).

### Mass-spectrometry

**Figure 3:**
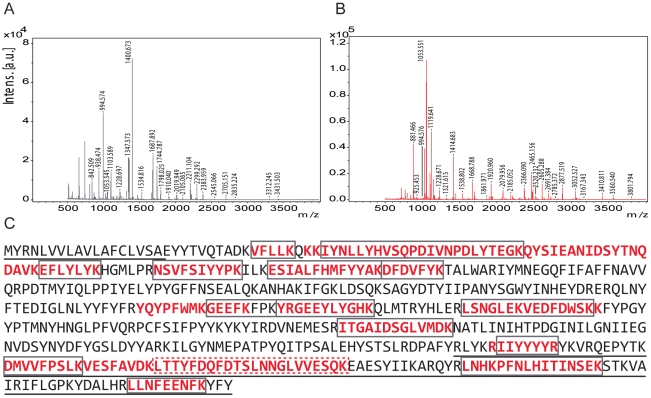
Mass-spectrometric spectra of Hexamerin 1 and Hexamerin 2, and the peptide hits of Hexamerin 2 against hexamerin sequence. The signal sequence in the N-terminus and the dsRNA in the C-terminus are underlined. A: MALDI-TOF/TOF spectra for Hexamerin 1. B: MALDI-TOF/TOF spectra for Hexamerin 2. C: MALDI-TOF/TOF peptide hits (grey boxes) and LC-MSMS hits (red text) for Hexamerin 2. A specifically inspected LC-MSMS peptide found in Hexamerin 2 data, is boxed with a red dashed line.

MALDI-TOF/TOF and LC-MSMS experiments revealed that Hexamerin 2 corresponds to the cloned gene based on 30%–40% sequence coverage, respectively (Fig. 3C). Hexamerin 1 had four hits against the sequence (YRGEEYLYGHK, LSNGLEKVEDFDWSK, RIIYYYYR and EASYIIKAR; 8% sequence coverage), but got no hits in the subsequent LC-MSMS analysis. Both protein samples also contained unidentified non-trypsin, non-keratin peptides. MASCOT search score [35] for Hexamerin 2 LC-MSMS result against the cloned sequence was 207, with a sequence-based emPAI [36] score of 1.20. The peptide LTTYFDQFDTSLNNGLVVESQK that spanned the region of the dsRNA was, in particular, identified for Hexamerin 2 (Fig. S2).

### Deglycosylation and phosphorylation

**Figure 4:**
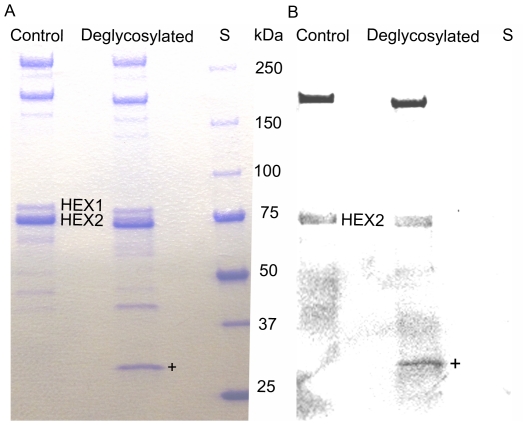
Glycosylation and phosphorylation of *P. metricus* (wild type) hexamerins. Both hexamerins are glycosylated, but only Hexamerin 2 is phosphorylated. **A:** Both hexamerins shift in size toward lower molecular weight after deglycosylation with PNGase F. **B:** The same gel stained with phosphorylation specific Pro-Q diamond stain (Invitrogen). Hexamerin 2 and vitellogenin (between 150 and 250 kDa standard bands) appear to be phosphorylated in *P. metricus* hemolymph. The + sign marks the deglycosylation enzyme PNGaseF.

The two hexamerins have glycosylation in common, but they differ in the case of phosphorylation. Both Hexamerin 1 and Hexamerin 2 show reduced molecular weight on SDS-PAGE after treatment with PNGase F that removes N-linked glycogroups (Fig. 4A). The estimated size-shift was approximately 1.6 kDa for Hexamerin 1 and 1.8 kDa for Hexamerin 2, likely corresponding to the size of the glycogroup or total glycogroups attached to these proteins. Staining with phosphorylation-specific stain showed that of the two putative hexamerins only Hexamerin 2 was phosphorylated, and it was the only major phosphorylated protein in addition to a putative vitellogenin in *P. metricus* hemolymph (Fig. 4B). This difference at the post-translational level might reflect the divergent effects of the proteins on *P. metricus*.

### Verification of hexamerin 2 down-regulation

**Figure 5:**
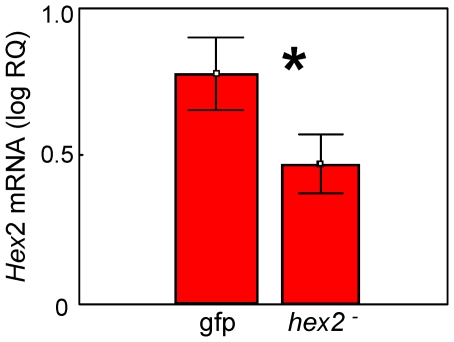
Validation of *hexamerin 2* knockdown after larval feedings. RNA isolated from whole larvae (n = 18 per treatment type) was used for qRT-PCR. Compared to controls, *hexamerin 2* dsRNA feeding elicited a significant reduction in *hexamerin 2* transcript. Bars represent means ± s.e.; asterisk = *P*<0.05.

Compared to controls, larvae fed *hexamerin 2* dsRNA (*hexamerin 2* RNAi) showed a significant reduction in *hexamerin 2* transcript levels (main affects ANOVA: F _1,32_ = 6.258, p = 0.018) (Fig. 5).

### RNAi phenotype and trait associations

Most of the 128 treated larvae (Table S2) could not be included in the final analysis. Forty-seven treated larvae were males, and 31 were cannibalized. Cannibalism occurs naturally and increases in frequency as colonies advance toward the end of their cycle [37]. Two colonies were destroyed (one by a mowing machine; one by *Solenopsis invicta* fire ants) for a loss of 10 specimens (6 *gfp*; 4 *hexamerin 2*). Eight larvae failed to feed or to spin cocoons. Because this is the first experiment of its kind we do not know if this was in response to treatments *per se*, disturbance during treatments, or some other cause. Seven larvae spun cocoons before receiving 4 treatment feedings (3 *gfp*; 4 *hexamerin 2*), and three adult wasps escaped. The remaining 22 larvae received 4 treatment feedings, spun cocoons, emerged as adult females, and were placed in the bioassay (11 total *gfp* from 5 nests; 11 total *hexamerin 2* from 4 nests). We used these 22 specimens (Table S3) for all treatment analyses.

Study site did not affect the wasps' phenotype (F_1,16_ = 1.000, P = 0.500, N = 5, 15). Treatment was also not a significant factor in this corrected model that blocked for the effect of location on phenotypic traits (F_1,16_ = 0.040, P = 0.874, N = 10). No interaction was detected between study site and treatment (F_1,16_ = 0.042, P = 0.840). Next, we took into account that the total nine nests were split between the treatment categories. Using a nested design ANOVA to control for effects of nest within treatment, we found that neither treatment (F_1, 13_ = 0.008, P = 0.929, N = 11) nor nest (F_7,13_ = 0.528, P = 0.798, N = 4, 5) was significant. Thus, although the phenotypes showed some variability between nests (one-way ANOVA: F_4, 20_ = 34.116, P = 0.023, N = 6, excluding three nests with only 1 individual observation), the treatment groups were equally affected by inter-individual heterogeneity. We concluded that putative effects of treatment were not confounded by environmental influences or heterogeneity. To conserve degrees of freedom, therefore, factors location and nest were not used for blocking in subsequent analyses.

We initially used a full, parametric model to analyze the phenotypic dataset. Total developmental time, ovary development, forewing length, and adult feeding were included as dependent variables in a one-way analysis of variance (ANOVA). Total developmental time was summed over the measurements of days to cocoon and days cocooned to avoid a colinearity problem caused by the two variables being significantly negatively associated in the dataset (Pearson correlation, R = −0.538, P = 0.001, N = 22). Egg number was not included due to insufficient data and variability (1 egg was laid by a *gfp* control wasp). The ANOVA suggested that *hexamerin 2* RNAi did not alter the *P. metricus* phenotype. This result was independent of whether ovary development was coded as a binary variable (i.e., ovary being active or not: F_4,17_ = 2.259, P = 0.105, N = 11) or as 4 developmental classes (F_4,17_ = 1.961, P = 0.146, N = 11). The analysis conformed to assumptions of ANOVA, as determined by Bartlett's test and Levene's test on variances and normality plots of residuals.

**Figure 6:**
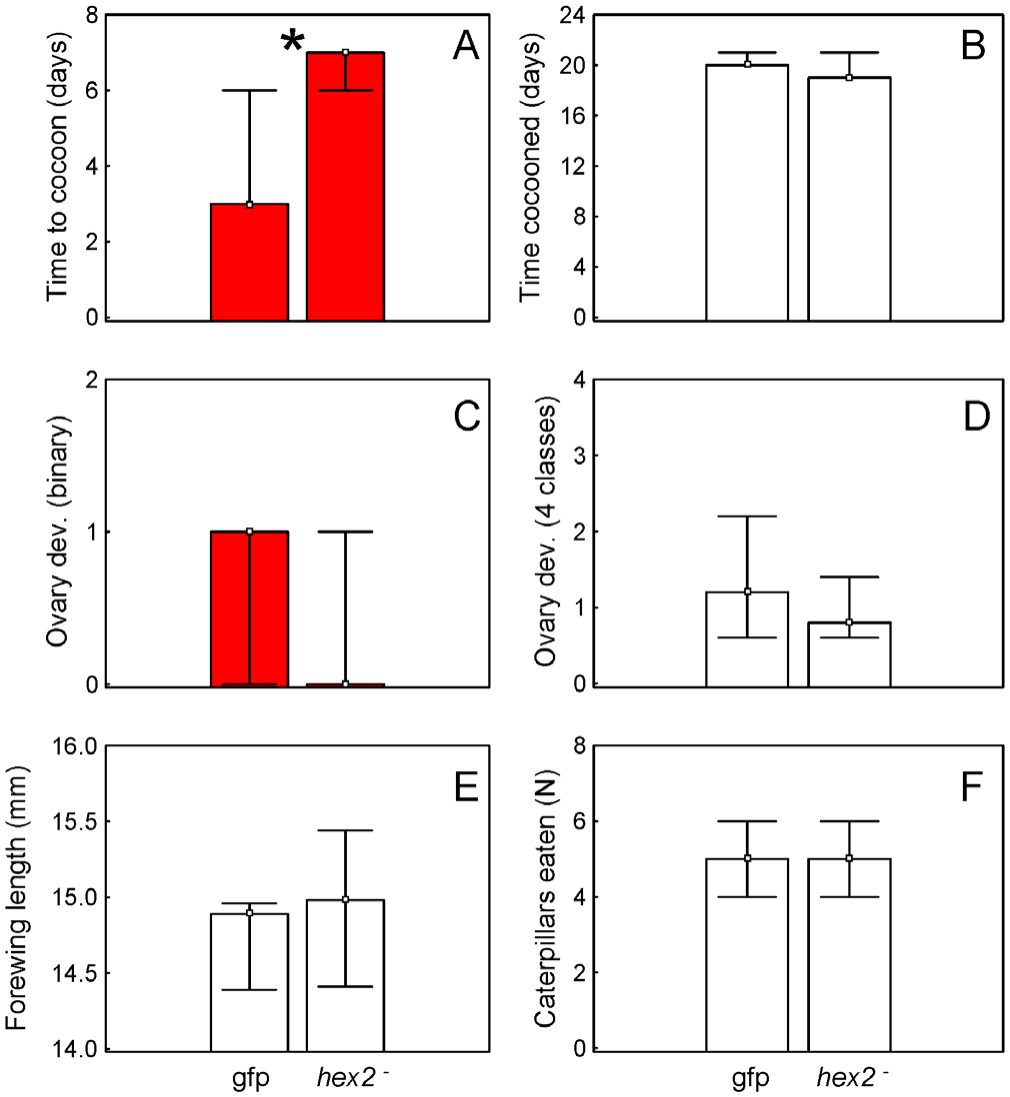
Median and 25–75% interquartile ranges for RNAi phenotypes. *P. metricus* larvae were assigned to a control group that received dsRNA for green fluorescent protein (*gfp*) or to a treatment group that received dsRNA against the *hexamerin 2* gene to trigger RNA interference (*hexamerin2 ^−^*). The wasps were monitored during post-treatment development and isolated for bioassays on the day they emerged as adults. **A:** The number of days from the start of treatment with dsRNA until cocoon spinning (time to cocoon). The significance indicator denotes Mann-Whitney U-test, P = 0.017, N = 11. This test statistic is significant only if multiple testing is not accounted for. **B:** The number of days between cocooning and adult emergence (time cocooned). **C, D:** Ovary development in 13 day-old adults as (C) a binary score (active or not – categories 1 and 2 of Fig. S3) and (D) scored as the 4 developmental classes of Figure S3. **E:** Length in mm of adult forewings from the proximal convergence of the C cell to the distal posterior corner of the 2Rs cell. **F:** Number of caterpillars eaten during the post-emergence isolation period. Control *gfp* and treatment *hexamerin2* ^−^ phenotypes differed significantly for time to cocoon.

Next, we looked at each variable using a non-parametric approach. We found that days cocooned, ovary development (4 classes), forewing length, and adult feeding were similar between *hexamerin 2* knockdowns and *gfp* controls (Mann-Whitney U-test, P>0.05, N = 11, Fig. 6B, D–F). In contrast, days to cocoon and ovary development score (binary) were significantly different and suggestively different, respectively (Mann-Whitney U-test, P = 0.017; P = 0.094, N = 11, Fig. 6A, C). These results suggest that *hexamerin 2* down-regulation could extend larval development and tentatively reduce ovarian development in adults. However, when corrected for multiple comparisons (Dunn-Sidak Bonferroni, alpha<0.009), neither effect could be considered as statistically reliable.

Finally, we studied the phenotypic trait associations independent of treatment group identity. Adding to the significant negative correlation between days to cocoon and days cocooned (above), the data showed a significant negative relationship between total developmental time and ovary development (Pearson correlation, R = −0.526, P = 0.012, N = 22). This finding indicated that, in the dataset overall, individuals with a shorter total developmental time were more likely to have developed ovaries when scored as adults.

## Discussion

Here we have reported the successful development of an RNA interference tool and characterization of its target protein for the primitively eusocial paper wasp *Polistes metricus*. Our most significant contribution to RNAi studies is that we then successfully deployed the RNAi tool in a field setting – that is, in a natural setting outside the laboratory. We did so in the context of an ecological test of caste differentiation in the eusocial wasp *Polistes*, which is a model system for evolutionary studies of insect sociality and behavioral ecology. The foundational aspects of our work included sequencing the *hexamerin 2* gene, matching the cloned gene to the Hexamerin 2 protein product, and identification of the correspondence between the cloned gene and Hexamerin 2 by mass-spectrometry. Our principal biological finding – that Hexamerin 2 plays no role in worker/gyne differentiation – coincides with findings from previous studies using other investigative methodologies.

Our research underscores that *Polistes* is a robust model system, having a rare convergence of assets. Paper wasps, with their 2 dimensional, bare nests allow access for observation and manipulation in their natural environment. As a result, they are a model that can be used to address questions regarding the structure and evolution of insect sociality. Most other social insects have been studied in artificial habitats. Ants and termites nest underground or within solid structures, thus making in-nest observations difficult or impossible in the field. Honey bees, although easily observed and the target of the first insect genome project, are domesticated animals and have been selected for docility and honey production. Our work combines the assets of the *Polistes* model system with tools of genomics deployed in a field setting, augmenting the studies of Toth et al. and Hunt et al. [17], [31], [38], [39] and further advancing the *Polistes* model system.

Our small final sample sizes (11 *hexamerin 2* and 11 *gfp*) could reflect several variables that can be addressed in future RNAi experiments. In order to provide maximum assurance that we treated only gyne-destined larvae, we conducted our experiment in late July and August, corresponding to the mid to late phase of gyne production. Because nest mortality is a normal demographic occurrence, a higher sample size could be obtained by beginning an experiment earlier in the phase of gyne production. To increase the number of nest boxes at the start of a season could also increase the final sample size. The frequency of nest foundings in our nest boxes by *P. metricus* (47/300) was unexpectedly lower than that of previous studies conducted in Missouri, USA.

The four putative Hexamerin 1 peptide hits (8% sequence coverage) toward the Hexamerin 2 sequence in the MALDI experiment could be explained by some level of contamination or by sequence identity in these regions. Because the gene that encodes Hexamerin 1 is not identified, precise sequence similarity estimation between two hexamerins is currently infeasible. Previous studies of other hymenopteran species with sequenced genomes have reported relatively high sequence similarity of multiple hexamerins: up to 42% in *A. mellifera* [40] and 79% in the wasp *Nasonia vitripennis* [41].

We found that the two putative hexamerins of *P. metricus* are glycosylated. In Hexamerin 2, the putative N-glycosylation sites are located at asparagines 201 and 344. Glycosylation has been shown to have important stabilizing and structural roles in a related storage protein, arylphorin, in *Antheraea pernyi* silk moth [42]. Based on SDS-PAGE size-estimation, Hexamerin 1 (∼77.7 kDa) lies within the typical hexamerin size scale of 75–90 kDa, whereas Hexamerin 2 is smaller than an average hexamerin with its molecular weight of ∼68.5 kDa [40], [41]. In addition to differences in size, only Hexamerin 2 appears to be phosphorylated. Phosphorylation can be critical for events ranging from signaling to subcellular protein localization [43], [44]. Hexamerin-receptor interaction is regulated by receptor phosphorylation in rice moth *Corcyra cephalonica* [45], but effect of phosphorylation of hexamerin itself are currently unclear. The differences that we found between Hexamerins 1 and 2 in both size and phosphorylation suggest different roles for the two proteins.

In a previous study using gel band quantification, Hexamerin 1 was significantly differentially expressed between worker-destined and gyne-destined larvae, but that experiment indicated no specific caste-related role for Hexamerin 2 [31]. In the present study we used dsRNA for the *hexamerin 2* gene to specifically down-regulate production of Hexamerin 2 protein. The absence of a significant caste-related response to RNAi down-regulation of Hexamerin 2 confirms the inference of the previous study. However, our detailed statistical analysis suggests possible trends toward extended larval development time and reduced ovary development in knockdowns, which are characteristics of gynes [31], [39]. The detailed statistical analysis is warranted, even though the statistical results *per se* are negative, because both positive and negative statistical outcomes are meaningful for interpretation of the study.

Whereas dsRNA injection studies achieve significant knockdown of the target gene at low dosage [46], [47], feeding studies generally require a higher dosage to achieve efficient knockdown. Effective knockdown in *Polistes* was achieved using a dosage of dsRNA consistent with feeding studies in honey bees [21], [48] and termites [22]. Control (*gfp*) and *hexamerin 2* larvae were fed equally with dsRNA without adverse phenotypic effects, thereby suggesting that the system will be compatible with future RNAi experiments. A next step, therefore, would be to continue investigation of roles played by hexamerins in *P. metricus* worker/queen differentiation using *hexamerin 1* RNAi in a full replication of the study we report here, once transcripts for the *P. metricus hexamerin 1* gene have been identified. Such an investigation could test the possibilities of compensatory or opportunistic upregulation of Hexamerin 1 by Hexamerin 2. Compensation would be physiological – to have Hexamerin 1 substitute for Hexamerin 2 in functions that are not yet known. ‘Opportunistic’ would be that Hexamerin 1 increases because more building blocks are available if less Hexamerin 2 is reduced. In addition, methods such as microarray [38] and RNAseq can be employed to identify candidate genes for other ecologically and evolutionarily important traits such as behavioral dominance, seasonal and nutritional affects on the colony cycle, photoperiodicity, and diapause. The genetic foundations of these traits can then be pursued using RNAi in experiments such as we report here. Ever-lower costs of whole genome sequencing using high throughput sequencing techniques, combined with improvements in annotation and comparative analysis methods, now allow genomic tools to be developed for many species. Efforts are currently underway to sequence genomes of two *Polistes* species: *P. dominulus* and *P. canadensis* (A. L. Toth, personal communication). Thus, soon there will be a substantial increase in genome sequence information for *Polistes*. The RNAi tool that we present here, therefore, is an extremely timely step forward that can enhance the use of paper wasps as model systems for the greater synthesis among gene expression, behavioral ecology, and evolutionary biology of social insects. In addition, although there are unique features of *Polistes* biology that make it particularly suitable for RNAi experiments in the field, our work suggests the possibility that RNAi is a tool that can be adapted for experiments on other species in natural environments.

## Methods

### Hexamerin sequencing

Four partial EST sequences for hexamerin (contigs 45616, 45913, 42334 and 40833: http://stan.cropsci.uiuc.edu/454/blast/waspblast.html) were available from the *Polistes* wasp EST data set to design primers. Total RNA isolated from 5^th^ instar larvae was treated with DNaseI (Ambion), and 5′ and 3′ RACE were carried out using the GeneRacer Kit (Ambion). For cloning, we used different combinations of primers for 5′ and 3′ RACE (Table S1) in conjunction with the RACE primers provided in the kit. Following PCR, the amplified products were separated on 1% agarose gel, and DNA was extracted using the QIAquick Gel Extraction Kit (Qiagen). Thereafter, the PCR products were cloned into pCR® 4-TOPO® vector using the TOPO TA cloning kit (Invitrogen). Several clones were randomly picked and verified by sequencing.

### MALDI-TOF/TOF

We identified the cloned hexamerin using peptide mass fingerprinting. To do so we ran dilution series of 0.2–0.8 µl *P. metricus* hemolymph from an adult gyne, a rich source of hexameric storage proteins [49], on a 4–20% gradient SDS-PAGE gel (Bio-Rad) followed by staining proteins with Coomassie blue (Bio-Rad). We then excised protein bands corresponding to Hexamerin 1 and Hexamerin 2 as identified by molecular weight [49] plus a clear gel region between the two proteins as a blank (control) sample. The samples were digested with trypsin and prepared for Ultra-flex MALDI-TOF/TOF (Bruker Daltonics) analysis according to existing protocols [50], [51] using nC18 StageTip microcolumns [36]. We then processed spectra with FlexAnalysis (Bruker Daltonics) and targeted the annotated peaks for peptide mass fingerprint analyses with Mascot (Matrix Science; in house Mascot server) using all the NCBI databases and the *P. metricus* hexamerin sequence information we obtained by RACE.

### LC-MSMS

We excised gel bands of Hexamerin1, Hexamerin 2 (4 gel pieces), and a blank sample as above. Peptides were prepared as above and separated by Ultimate 3000 nano-HPLC (Dionex Corporation, USA) using a nC18 reverse phase column coupled to a 4000 Q-Trap mass spectrometer (Applied Biosystems/MDS SCIEX, Concord, ON, Canada) and eluted as described elsewhere [51]. We used Analyst (AB SCIEX) for data processing and Mascot for data analysis. Mascot search was done against the *P. metricus* hexamerin sequence embedded in all the common NCBI databases. We set the significance threshold at 0.05, ion score minimum at 20, and included only hits with expected values <0.1. A putative peptide corresponding to the size of the sequence LTTYFDQFDTSLNNGLVVESQK, located at the dsRNA region, was inspected in detail in the elution pattern of the Hexamerin 2 peptides.

### Deglycosylation and staining of phosphorylated proteins

We tested Hexamerin 1 and Hexamerin 2 for post-translational modifications, glycosylation and phosphorylation, to gain protein-level insight and to make a comparison between the hexamerins. In order to test the presence of N-linked glycogroups, we treated 0.2 µl *P. metricus* hemolymph for 1 h at 37°C with 1 µl PNGase F and kit buffers (New England BioLabs), followed by a 10 min denaturation at 95°C. We treated a control sample similarly but with no PNGase F. We then ran samples on a 4–20% gradient gel (Bio-Rad) and stained them with Pro-Q Diamond (Invitrogen), which exclusively stains phosphorylated proteins. Finally, we visualized the gel in trans-UV light with Gel Doc Imager (Bio-Rad) prior to staining it with Coomassie blue (Bio-Rad) for detection of all proteins.

### dsRNA synthesis

dsRNA was synthesized as described by Amdam et al. [24] using the AmpliScribe T7 transcription kit (Epicentre Biotechnologies) for bidirectional *in vitro* transcription of a plasmid DNA construct using gene specific primers with flanking T7 transcription initiation sites. For *Hexamerin 2* dsRNA synthesis, we used the following forward and reverse primers fused with the T7 promoter sequence (underlined): 5′-TAATACGACTCACTATAGGGCGACGGCTCTACAAGAGGATAATC-3′ and 5′-TAATACGACTCACTATAGGGCGAGAAATATTTGAAGTTCTCTTC-3′. The resulting dsRNA product was 360 bp long. As an RNAi treatment control, *gfp* dsRNA, was synthesized using primers that cover 520 bases in the open reading frame of the AF097553 template as previously described for gene targeting in honey bees (Wang et al 2010). The dsRNA was purified using phenol∶choloform extraction and verified for size and purity by running on 1% agarose gels. The final concentration of dsRNA was 4–5 µg/µl. The sequence used to generate *hexamerin 2* dsRNA is underlined in Figure 2.

### Larval sex determination

We determined larval sex by genotyping 9 microsatellite loci that were previously developed from expressed sequence tag sequences [52]. We used 2 µg of total RNA for cDNA synthesis using SuperScript VILO cDNA Synthesis Kit (Invitrogen) and then used the cDNA for microsatellite genotyping. PCR reactions and fragment visualization were performed as previously described [31].

### Verification of hexamerin 2 knockdown

Fifth-instar larvae received 2 feedings per day for 2 days of either *hexamerin 2* dsRNA or *gfp* dsRNA. Two days later, in the field, we processed single specimens as each was removed from its nest cell. Larval gut contents, called meconium, are contained within a chitinous peritrophic sac. The meconium volume is particularly large in fifth-instar larvae. To remove the meconium makes subsequent processing of the specimen easier and also obviates any possible RNA degradation. Using a pointed scalpel, we pierced the body and midgut walls and removed the meconium by grasping the peritrophic sac with fine-tipped forceps and quickly extracting it. The larva was then flash frozen in liquid N_2_. Specimens were further processed in the lab by homogenization and storage in Trizol reagent. We randomly selected 18 female larvae per treatment group for knockdown verification via real-time quantitative reverse-transcriptase PCR (qRT-PCR) using a one-step qRT-PCR kit (Ambion). Total RNA was isolated and treated with turbo DNase I (Ambion). Briefly, total RNA (25 ng/µl) was used as the template for reverse transcription. All qPCR reactions were run in triplicate using an ABI Prism 7500 (Applied Biosystems), and we analyzed the data with the Delta-Delta CT method [53] with *rps8* as reference gene [17], [54]. The *hexamerin 2* forward and reverse primers were 5′-AAACAGCATTATGGGCTCGT-3′ and 5′-GACGTTGAACGACAGCATT-3′ respectively. For *rps*8, the forward and reverse primers were 5′-GAAGCGAAAGCCTCTCAGAA-3′ and 5′-AGGCGCAGTGCTCTGTATTT-3′. A negative control (without reverse transcriptase enzyme) was run for every sample to monitor DNA contamination. We used melt curve analysis to verify the absence of primer-dimers.

### Field rearing and in vivo dsRNA feedings

**Figure 7:**
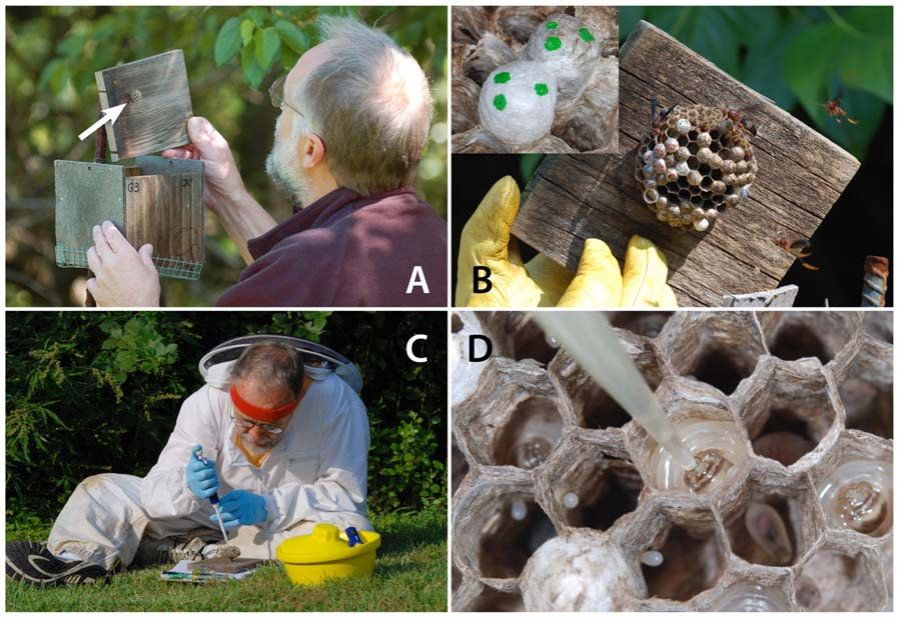
Field methods and dsRNA delivery. **A:** “Wasp boxes” have lift-off lids and wire mesh across the open bottom. Boxes were put in place in springtime prior to natural nest establishment on the undersides of lids by *P. metricus* foundresses. The early foundress phase nest in this box is indicated by the arrow. **B:** A reproductive phase study nest on the underside of a box lid during the experiments. Newly-spun cocoons of larvae that had received dsRNA were marked with dots of paint (inset). **C:** dsRNA and sucrose solutions were carried to the field on ice and mixed immediately prior to larval treatments. **D:** dsRNA/sucrose solution was fed directly to the mouthparts of large 5^th^-instar larvae via micropipette. Larvae consumed the sample quickly, generally in ∼1 sec.

We reared and monitored colonies of *P. metricus* using established protocols [30], [55], [56] (Fig. 7A). We placed three hundred nest boxes in weedy fields and at the margins of lawns and hay fields, all of which were near woodlands at sites owned and managed by North Carolina State University or the North Carolina Department of Agriculture in Wake Co., NC, within 10 km of the North Carolina State University (NCSU) campus. Forty-seven *P. metricus* nests were founded, all but one by a single foundress each. We worked only with single-foundress colonies. During the course of the field work we monitored colony development daily by removing the nest box top (Fig. 7B) and recording the content of each nest cell (empty, egg, larval instar, or cocoon) on a ‘nest map’ drawn on hexagon-imprinted paper. We collected colony data in the early mornings, generally before 9 am EDT, which enabled counting the number of adults present before daily foraging had begun.

We conducted all dsRNA treatments in the field using late 5^th^ instar larvae in single-foundress colonies *in situ* in the nest boxes. Hexamerins 1 and 2 are not expressed in 1^st^ through 4^th^ instar larvae [49]. Instead, they are expressed in 5^th^ (final) instar larvae and reach peak abundances at the larva to pupa molt [31], [49]. Our RNAi treatments were applied to late 5^th^ instar larvae to coincide with peak Hexamerin expression, thus our results are not a consequence of having treated larvae in a time window that might not yield a response signal.

Only a single dsRNA type was used per nest. For treatments, tubes of dsRNA were placed on ice and transported to the field, where we mixed the dsRNA with 30% sucrose (Fig. 7C) immediately before feeding it to larvae using a standard laboratory pipette (Fig. 7D). At each feeding we gave each larva 15 µl of the dsRNA/sucrose mix, which they readily accepted. Each larva was treated morning and evening on 2 consecutive days. Each feeding contained 20 µg of dsRNA, therefore a total of 80 µg of dsRNA was administered to each larva. A total of 72 larvae from 10 nests were fed *hexamerin 2* dsRNA, and a total of 56 from 8 nests were fed *gfp* dsRNA (Table S2). We identified larvae by location on the nest map. When a fed larva completed growth and spun its cocoon, we marked that cocoon with dots of Testors® enamel (Fig. 7B inset). We continued nest mapping daily until all fed larvae in a nest had spun cocoons.

When all fed larvae in a nest had spun cocoons, we transferred that colony to a rearing cage as described in [57]. Adults taken with the nest were anesthetized with CO_2_ and examined for wing wear. From 1 to 5 females with wing wear, which characterizes foundresses and workers [39], were marked with a dot of enamel on the metanotum and caged with the nest. We released any males and females with unworn wings, which were putative gynes [39]. We removed and discarded all unmarked cocoons and the untreated developing wasps in them as well as any subsequently cocooning individuals. Eggs and pre-cocooning larvae were undisturbed. We held caged colonies in a sheltered outdoor location at the NCSU Honey Bee Research Facility, where each colony was provided with *ad libitum* water and sucrose (rock candy) and a fresh mid-3^rd^ instar *Manduca sexta* caterpillar daily. When all treated wasps had emerged from a nest, the remaining colony was returned to its nest box in the field and thereafter not disturbed. The field studies did not involve any endangered or protected species. No specific permits were required for the described field studies.

### Treatment bioassay

We inspected caged colonies daily at mid-morning and identified newly-emerged wasps by nest map location prior to transporting them to the laboratory and placing each in an isolation cage. Isolation cages had wooden tops and bottoms ∼8.5 cm on each side. Three sides of the cage were wire mesh (∼3 mm openings) ∼23 cm in height. The fourth side was closed with corrugated cardboard into which a small doorway was cut for daily maintenance. A small, empty, pre-emergence nest (7 to 14 cells) constructed in the preceding year by a *P. metricus* foundress near St. Louis, MO, was affixed to the underside of the top of each cage before the newly-emerged wasp was introduced. Most wasps spent time on those nests.

Caged wasps were placed in growth chamber C25 in the NCSU Phytotron. The chamber contained incandescent lights on a 16L/8D cycle; fluorescent lights came on 2 h following and went off 2 h before the incandescent lights. Light intensity was 21 micromoles/sec/m^2^ in the incandescent-only morning and evening periods and 225 micromoles/sec/m^2^ during the both-lights midday. Chamber temperature was 20°C at night and 30°C during full light, with a gradual ramp-up in the morning and ramp-down in the evening. We provided each isolation cage with a small plastic Petri dish containing sucrose (rock candy), a water source (a 1.5 ml microfuge tube plugged with cotton), and, daily, a single small *M. sexta* caterpillar: late 2^nd^-instar or early 3^rd^-instar. Caterpillar head capsules were crushed immediately before provisioning, which ensured that they did not crawl away. Cage maintenance, conducted daily at midday, consisted of replenishing the water (deionized), removing the remains of any uneaten caterpillars, and providing a fresh caterpillar. Time outside the chamber was no more than 2–3 min. Each day we repositioned cages within the chamber.

### Treatment response variables

In a previous laboratory experiment with newly-emerged isolated *P. metricus* females, putative workers showed ovarian development whereas putative gynes did not [11]. Wasps entering ovary development could be expected to show higher rates of feeding from protein sources than wasps not entering ovary development. Because ovary development occurs in putative workers but not putative gynes, and because ovary development could reflect post-emergence feeding, we quantified both feeding rate and ovary development in order to test whether *hexamerin 2* treatment might induce ovaries more worker-like than those of controls. Beginning the day after being introduced to the chamber, we scored each wasp daily for 12 days as to whether the caterpillar had been eaten. On the 13^th^ day we removed each wasp from the chamber, chilled it ∼10 min in a refrigerator, and removed it from its cage.

We dissected ovaries (together with the digestive tract) by grasping the terminal segment of the metasoma with fine-point forceps and pulling. Removed tissue was placed in 100% EtOH in a labeled microfuge tube and stored at −20°C. Approximately 2 weeks after the last specimen had been prepared, we transferred the tissue samples to 70% EtOH and returned them to −20°C. Approximately 2 months later we dissected the tissue specimens in 70% EtOH under a dissecting microscope to separate the ovaries from other tissues. We then placed the ovaries in 70% EtOH in number-coded 0.2 ml microfuge tubes and held them at laboratory ambient temperature. Approximately 1 month later we digitally photographed each ovary using an Evolution MP Color camera on an Olympus SZX12 microscope with a 1.0× objective lens and the adjustable magnification set at 25×. All images were captured in a single session with no adjustments to the apparatus.

Four ovary images were selected as representing 4 development classes: 0 = ovaries filamentous with no discernable oocytes; 1 = oocytes discernable but small in size and somewhat translucent; 2 = one or more oocytes opaque (with yolk deposition); 3 = one or more large ova present (Fig. S3). We removed the selected images from the image set, and the remaining images were independently scored 0 to 3 by five non-biologists who compared each image to printed copies of Figure S3. For every ovary, we used the mean of these 5 objective scores for analyses. Scorers were blind to the identities of the specimens.

We clipped the right forewing of each wasp at its base using iris scissors and taped it to a labeled microscope slide with clear tape. We then captured digital images of the wings with the same camera/microscope equipment as for ovaries but with a 0.5× objective lens and with the adjustable magnification set at 10×. All images were captured in a single session with no adjustments to the equipment. We quantified images using ImageJ software. A linear measurement (in pixels) was taken from the proximal closure of the C cell to the distal posterior corner of the 2Rs cell (nomenclature as in [58]). Wing wear by some wasps when in isolation precluded total wing length measurement. Measurements were blind with regard to which dsRNA a wasp had received. We converted pixel values to mm by quantifying a scale photographed at the same magnification.

### Statistical analyses

The nests that received *gfp* and *hexamerin 2* dsRNA treatment to verify *hexamerin 2* knockdown were equally represented at two study sites. For RNAi validation, we collected *gfp* control larvae from six nests and putative *hexamerin 2* knockdown larvae from four nests. Only a single dsRNA was administered in a single nest. Gene expression data were log transformed. We then used a main effects ANOVA in a model with mRNA level as the dependent variable and with qPCR plate (technical blocking factor) and treatment as the dependent variables. The dataset conformed to assumptions of ANOVA, as determined by Bartlett's tests and normality plots of residuals.

For analyses of individual development and adult bioassays, the *gfp* controls represented five nests at three locations, and the *hexamerin 2* dsRNA treatment group represented four nests and three locations. The majority of the data (20 out of 22 wasps) came from two overlapping sites. To test whether treatment effects could be confounded by environmental influences or heterogeneity between location and nests, we first tested the effect of these two study sites on phenotypic characteristics, using a general linear model with location as random factor, treatment as a fixed effect, and ovary development, total developmental time, forewing length, and number of caterpillars eaten as dependent variables.

All analyses were performed with Statistica 6.0.

## Supporting Information

Figure S1 **Amino acid alignment of the putative** ***P. metricus*** **hexamerin protein with other known insect hexamerin sequences.** *Polistes* sequence was aligned with sequences of from other hymenopteran insects species, *Apis meliferra* (ABR45904), *Nasonia vitripennis* (XP_001607029) and *Camponotus festinatus* (CAB62053), and dipteran insects, *Aedes aegypti* (XP_001663961), *Culex quinquefasciatus* (XP_001843494) and *Anopheles gambiae* (XP_321434) using ClustalW version 1.82. The hemocyanin_N, (residues 29–151), hemocyanin_M domains (residues 158–415), and the hemocyanin_C, (residues 435–519) are all under-lined with dashed, solid and double-dashed lines respectively. Conserved residues among all taxa are highlighted in black bars, conserved substitutions among all taxa are highlighted in gray bars and semi-conserved substitutions are highlighted by light gray bars. Gaps in the alignment are indicated by dashes.
(TIF)

Figure S2 **Electrospray mass spectrometric scan of Hexamerin 2 peptides.** Peptide corresponding to dsRNA sequence LTTYFDQFDTSLNNGLVVESQK with mass 840.4 Da, indicated with the arrow, is only found in Hex2.
(TIF)

Figure S3 **Ovary categories 0 to 4 that were used as comparators for scoring ovary development.**
(TIF)

Table S1 Primer sequences used for cloning *P. metricus hexamerin* 2.
(DOC)

Table S2 Data records for all treated specimens. Columns, left to right, are the specimen identifier, treatment, date of first feeding with dsRNA, date of the fourth (final) feeding with dsRNA, total number of feedings received, date on which the pupal cocoon was spun, number of days from the first feeding with dsRNA until the cocoon was spun, number of days from cocoon spinning until adult emergence, sex, and fate of the specimen.
(DOC)

Table S3 Specimen identities, dsRNA treatments, and bioassay response data for the 22 adult wasps used for all analyses.
(DOC)
