# Supplementary figures and images for: Development of an RNA Interference Tool, Characterization of Its Target, and an Ecological Test of Caste Differentiation in the Eusocial Wasp Polistes
Source: PLoS One. 2011 Nov 1;6(11):e26641. doi: 10.1371/journal.pone.0026641 (PMC3206021; doi:10.1371/journal.pone.0026641)

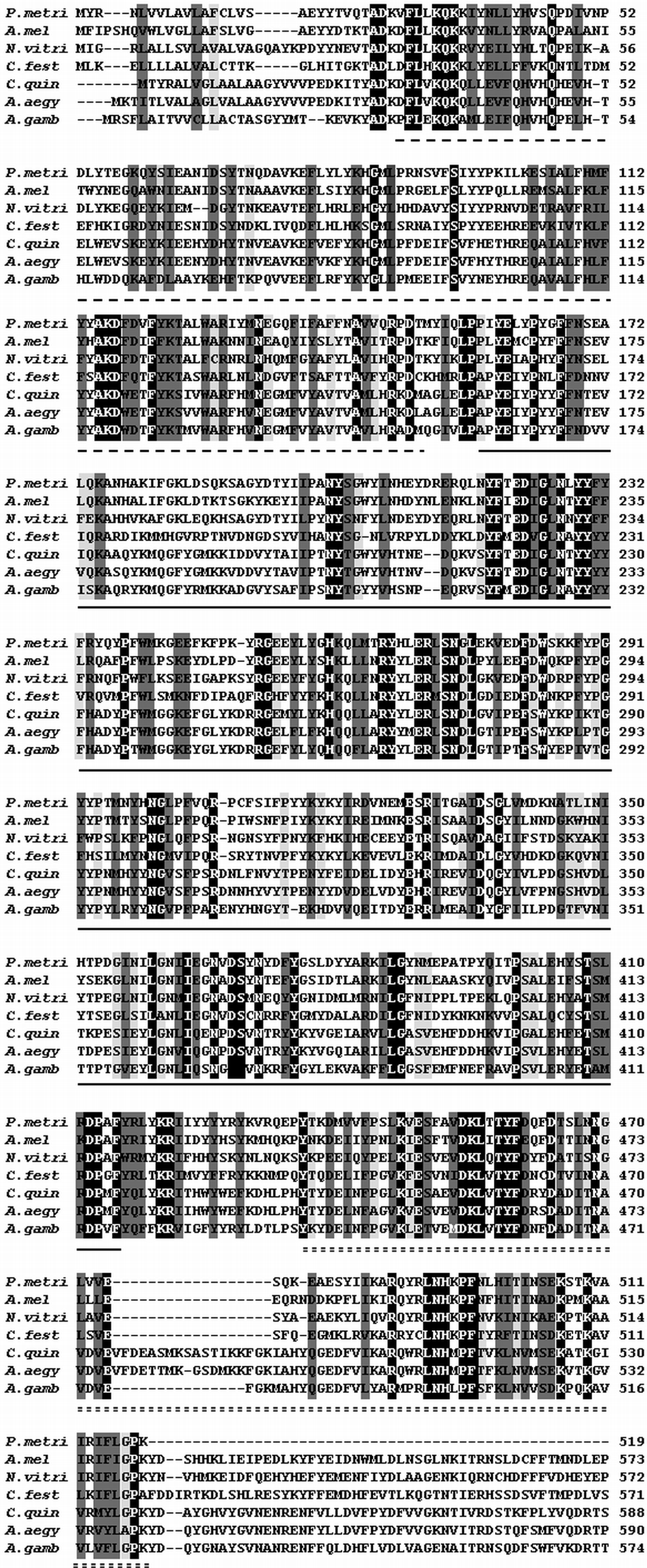

Supplement: Figure S1 — Amino acid alignment of the putative P. metricus hexamerin protein with other known insect hexamerin sequences. Polistes sequence was aligned with sequences of from other hymenopteran insects species, Apis meliferra (ABR45904), Nasonia vitripennis (XP_001607029) and Camponotus festinatus (CAB62053), and dipteran insects, Aedes aegypti (XP_001663961), Culex quinquefasciatus (XP_001843494) and Anopheles gambiae (XP_321434) using ClustalW version 1.82. The hemocyanin_N, (residues 29–151), hemocyanin_M domains (residues 158–415), and the hemocyanin_C, (residues 435–519) are all under-lined with dashed, solid and double-dashed lines respectively. Conserved residues among all taxa are highlighted in black bars, conserved substitutions among all taxa are highlighted in gray bars and semi-conserved substitutions are highlighted by light gray bars. Gaps in the alignment are indicated by dashes. (TIF) [file pone.0026641.s001.tif]

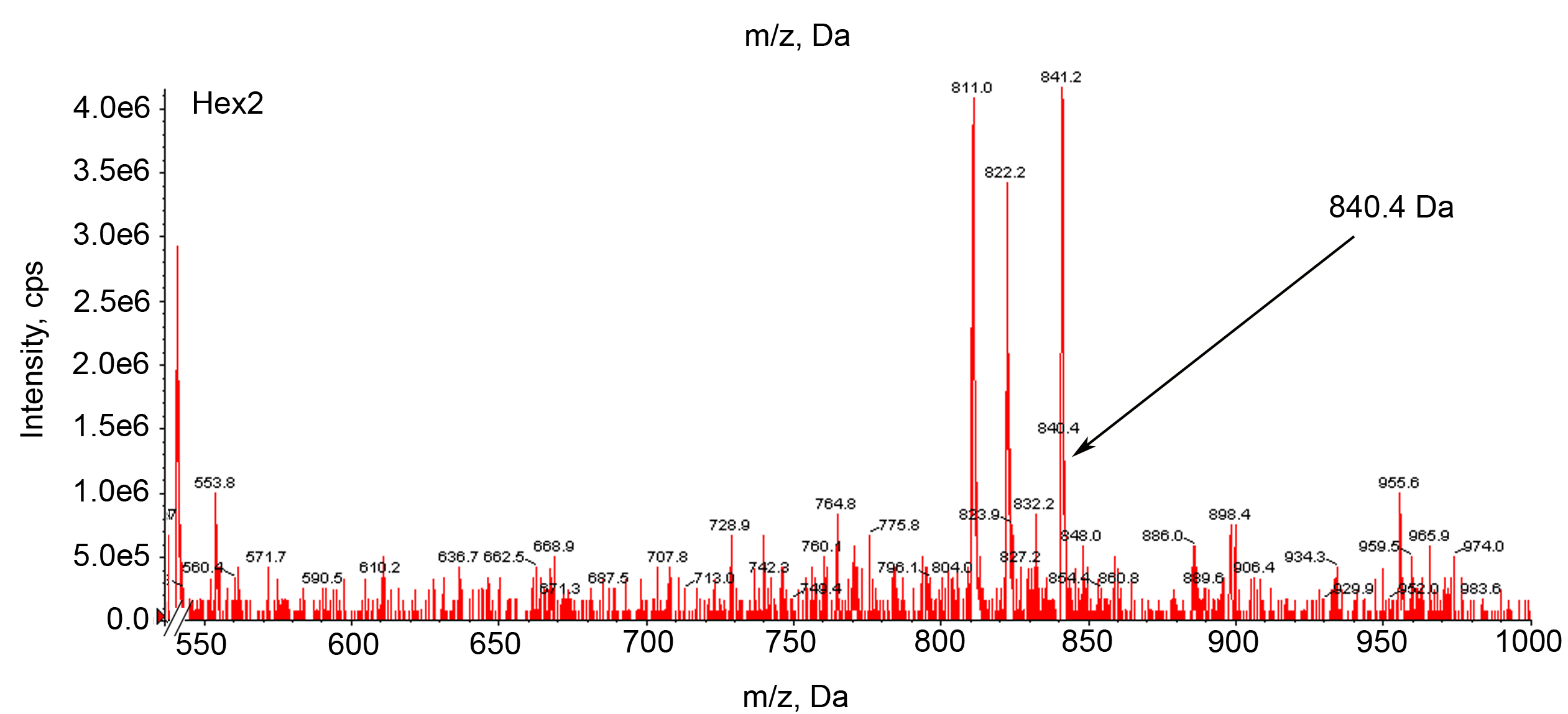

Supplement: Figure S2 — Electrospray mass spectrometric scan of Hexamerin 2 peptides. Peptide corresponding to dsRNA sequence LTTYFDQFDTSLNNGLVVESQK with mass 840.4 Da, indicated with the arrow, is only found in Hex2. (TIF) [file pone.0026641.s002.tif]

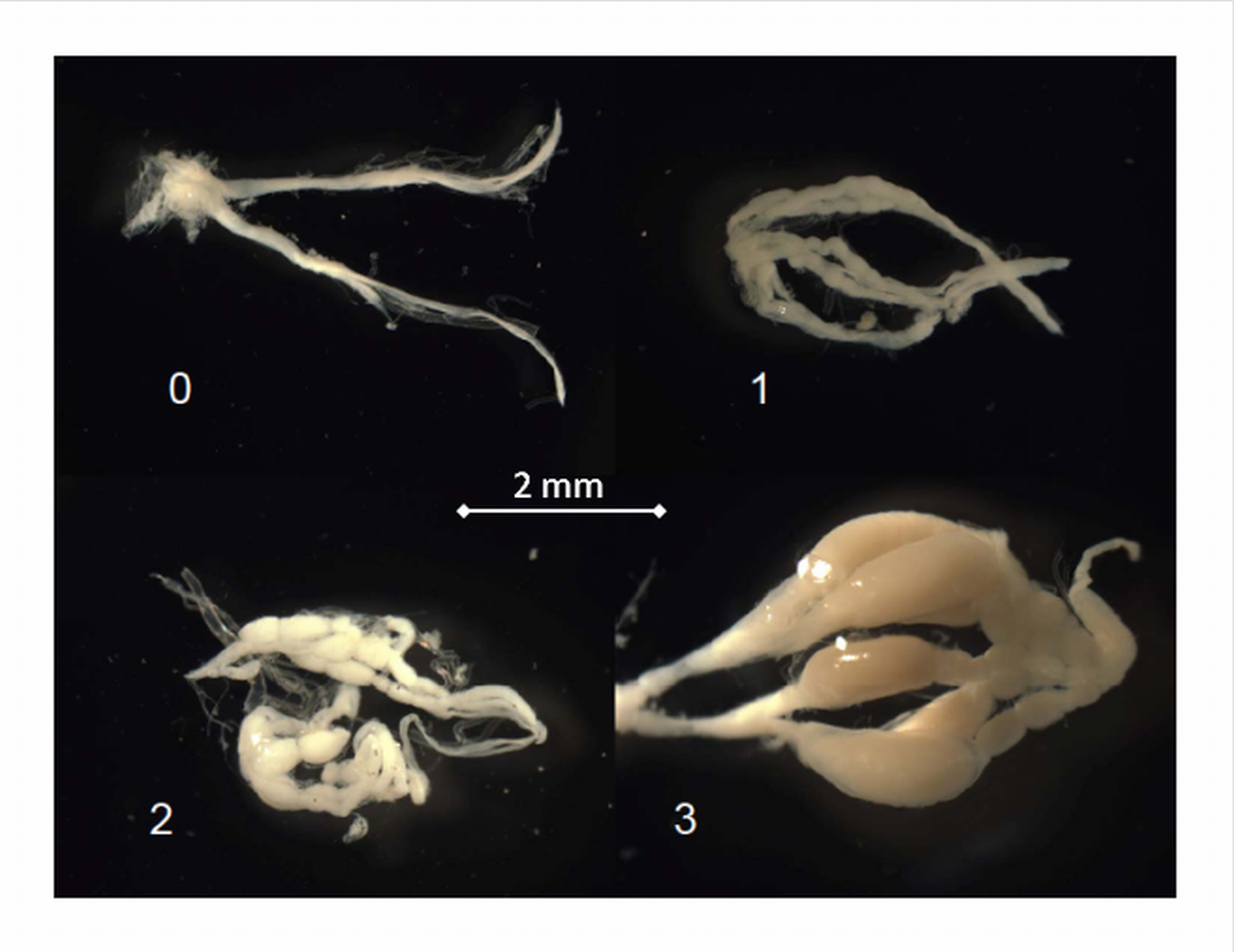

Supplement: Figure S3 — Ovary categories 0 to 4 that were used as comparators for scoring ovary development. (TIF) [file pone.0026641.s003.tif]
