# Supplementary material for: Development of an RNA Interference Tool, Characterization of Its Target, and an Ecological Test of Caste Differentiation in the Eusocial Wasp Polistes
Source: PLoS One. 2011 Nov 1;6(11):e26641. doi: 10.1371/journal.pone.0026641 (PMC3206021; doi:10.1371/journal.pone.0026641)
Supplement: Table S1 — Primer sequences used for cloning P. metricus hexamerin 2. (DOC) [file pone.0026641.s004.doc]

| 5’ RACE | Primer | Sequence |
| --- | --- | --- |
|  | Rp1 | 5’ TTATGGGTCCGAGGAAAATG 3’ |
|  | Rp2 | 5’ GTCCGAGGAAAATGCGAATA 3’ |
|  | Rp3 | 5’ AGCCAATATCTTCAGTGAAGT 3’ |
|  | Rp4 | 5’AGAGCCGATAAAAGCTGGA 3’ |
| 3’ RACE | Fp1 | 5’ AAGTCAGCTGGATATGACACA 3’ |
|  | Fp2 | 5’CAAATCACACCAAGTGCTCTTGAAC 3’ |
|  | Fp3 | 5’ ATCGGCTCTACAAGAGGATAATCT 3’ |
|  | Fp4 | 5’ AAGGCAAATCACGCAAAGAT 3’ |
|  | Fp4 | 5’ AGCTCTTCAGAAGGCAAATCAC 3’ |
|  | Fp5 | 5’ ACTTCACTGAAGATATTGGCT 3’ |
