# Supplementary material for: Development of an RNA Interference Tool, Characterization of Its Target, and an Ecological Test of Caste Differentiation in the Eusocial Wasp Polistes
Source: PLoS One. 2011 Nov 1;6(11):e26641. doi: 10.1371/journal.pone.0026641 (PMC3206021; doi:10.1371/journal.pone.0026641)
Supplement: Table S2 — Data records for all treated specimens. Columns, left to right, are the specimen identifier, treatment, date of first feeding with dsRNA, date of the fourth (final) feeding with dsRNA, total number of feedings received, date on which the pupal cocoon was spun, number of days from the first feeding with dsRNA until the cocoon was spun, number of days from cocoon spinning until adult emergence, sex, and fate of the specimen. (DOC) [file pone.0026641.s005.doc]

| Specimen | Treatment | 1st feed | 4th feed | # feedings | Cocoon date | Days to cocoon | Emerge date | Cocoon  Duration | Sex | Fate |
| --- | --- | --- | --- | --- | --- | --- | --- | --- | --- | --- |
| A16-1 | Hex | 20-Jul | 21-Jul | 4 | 26-Jul | 5 | 15-Aug | 20 | F | escaped |
| A16-2 | Hex | 20-Jul | 21-Jul | 4 | 28-Jul | 7 | 15-Aug | 18 | F | bioassay |
| A16-3 | Hex | 20-Jul | 21-Jul | 4 | 28-Jul | 7 | 16-Aug | 19 | F | bioassay |
| A16-4 | Hex | 20-Jul | 21-Jul | 4 | 28-Jul | 7 | 16-Aug | 19 | F | bioassay |
| A16-5 | Hex | 20-Jul | 21-Jul | 4 | 28-Jul | 7 | 16-Aug | 19 | F | bioassay |
| A17-1 | GFP | 20-Jul | 21-Jul | 4 | 29-Jul | 8 | 15-Aug | 17 | F | bioassay |
| A17-2 | GFP | 20-Jul | 21-Jul | 4 | 31-Jul | 10 |  |  |  | cannibalized |
| A17-3 | GFP | 20-Jul | 21-Jul | 4 | 31-Jul | 10 |  |  |  | cannibalized |
| A17-4 | GFP | 20-Jul | 21-Jul | 4 | 31-Jul | 10 |  |  |  | cannibalized |
| C6-1 | GFP | 2-Aug |  | 1 |  |  |  |  |  | cannibalized |
| C6-2 | GFP | 2-Aug | 3-Aug | 3 | 11-Aug | 8 | 1-Sep | 21 | M | male |
| C6-3 | GFP | 2-Aug | 3-Aug | 2 | 4-Aug | 1 | 24-Aug | 20 | F | <4 feedings |
| C6-4 | GFP | 2-Aug | 3-Aug | 2 | 4-Aug | 1 | 24-Aug | 20 | F | <4 feedings |
| C6-5 | GFP | 2-Aug | 3-Aug | 4 | 9-Aug | 6 | 30-Aug | 22 | M | male |
| C6-6 | GFP | 2-Aug | 3-Aug | 4 | 9-Aug | 6 |  |  |  | cannibalized |
| C6-7 | GFP | 2-Aug | 3-Aug | 4 |  |  |  |  |  | cannibalized |
| G40-1 | GFP | 6-Aug | 7-Aug | 4 | 8/9-Aug | 1 or 2 | 28-Aug | 20 | M | male |
| G40-2 | GFP | 6-Aug | 7-Aug | 4 | 12-Aug | 5 |  |  |  | cannibalized |
| G40-3 | GFP | 6-Aug | 7-Aug | 4 | 12-Aug | 5 |  |  |  | cannibalized |
| G40-4 | GFP | 6-Aug | 7-Aug | 4 | 13-Aug | 6 |  |  |  | cannibalized |
| G40-5 | GFP | 6-Aug | 7-Aug | 4 | 17-Aug | 10 | 6-Sep | 20 | M | male |
| G40-6 | GFP | 6-Aug | 7-Aug | 4 | 19-Aug | 12 |  |  |  | cannibalized |
| G51-1 | Hex | 6-Aug |  |  |  |  |  |  |  | did not feed |
| G51-2 | Hex | 6-Aug |  |  |  |  |  |  |  | cannibalized |
| G51-3 | Hex | 6-Aug |  |  |  |  |  |  |  | cannibalized |
| G51-4 | Hex | 6-Aug |  |  |  |  |  |  |  | did not feed |
| H1-1 | Hex | 20-Jul | 21-Jul | 4 | 22-Jul | 1 |  |  |  | cannibalized |
| H1-2 | Hex | 20-Jul | 21-Jul | 4 | 24-Jul | 3 | 12-Aug | 21 | F | escaped |
| H1-3 | Hex | 20-Jul | 21-Jul | 4 | 29-Jul | 8 | 19-Aug | 21 | F | bioassay |
| H1-4 | Hex | 20-Jul | 21-Jul | 4 | 28-Jul | 7 | 16-Aug | 19 | F | bioassay |
| H1-5 | Hex | 6-Aug | 7-Aug | 4 |  |  |  |  |  | cannibalized |
| H1-6 | Hex | 6-Aug | 7-Aug | 4 | 11-Aug | 4 | 3-Sep | 23 | M | male |
| H1-7 | Hex | 6-Aug | 7-Aug | 4 | 13-Aug | 6 | 4-Sep | 22 | M | male |
| H1-8 | Hex | 6-Aug | 7-Aug | 4 | 13-Aug | 6 | 1-Sep | 19 | F | bioassay |
| H1-9 | Hex | 6-Aug | 7-Aug | 4 | 13-Aug | 6 | 5-Sep | 23 | M | male |
| H1-10 | Hex | 6-Aug | 7-Aug | 4 | 13-Aug | 6 | 6-Sep | 24 | M | male |
| H1-11 | Hex | 6-Aug | 7-Aug | 4 | 15-Aug | 8 |  |  |  | cannibalized |
| H1-12 | Hex | 6-Aug | 7-Aug | 4 | 16-Aug | 9 | 3-Sep | 18 | F | bioassay |
| H8-1 | Hex | 28-Jul | 29-Jul | 3 | 29-Jul | 0 | 20-Aug | 22 | F | <4 feedings |
| H8-2 | Hex | 28-Jul | 29-Jul | 4 | 4-Aug | 6 | 27-Aug | 23 | M | male |
| H8-3 | Hex | 28-Jul | 29-Jul | 4 | 5-Aug | 7 | 29-Aug | 24 | M | male |
| H8-4 | Hex | 28-Jul | 29-Jul | 4 | 4-Aug | 6 |  |  |  | cannibalized |
| H13-* | GFP | 20-Jul | 21-Jul | 3 | 21-Jul | 0 | 13-Aug | 23 | F | escaped |
| H13-1 | GFP | 20-Jul | 21-Jul | 4 | 28-Jul | 7 | 16-Aug | 19 | F | bioassay |
| H13-2 | GFP | 20-Jul | 21-Jul | 4 | 28-Jul | 7 | 18-Aug | 21 | F | bioassay |
| H13-3 | GFP | 20-Jul | 21-Jul | 4 | 26-Jul | 5 | 15-Aug | 20 | F | bioassay |
| H16-1 | Hex | 28-Jul | 29-Jul | 4 | 2-Aug | 4 | 23-Aug | 21 | F | bioassay |
| H16-2 | Hex | 28-Jul | 28-Jul | 4 | 6-Aug | 9 | 28-Aug | 22 | M | male |
| H16-3 | Hex | 28-Jul | 28-Jul | 1 | 28-Jul | 0 | 19-Aug | 22 | M | male |
| H16-4 | Hex | 28-Jul | 28-Jul | 2 | 29-Jul | 1 | 20-Aug | 22 | F | <4 feedings |
| H16-5 | Hex | 28-Jul | 29-Jul | 4 | 31-Jul | 2 | 21-Aug | 21 | F | bioassay |
| H16-6 | Hex | 29-Jul | 29-Jul | 1 | 31-Jul | 2 | 21-Aug | 21 | F | <4 feedings |
| H19-1 | Hex | 4-Aug | 5-Aug | 4 | 8-Aug | 3 | 30-Aug | 22 | M | male |
| H19-2 | Hex | 4-Aug | 5-Aug | 4 | 8-Aug | 3 | 30-Aug | 22 | M | male |
| H19-3 | Hex | 4-Aug | 5-Aug | 4 | 9-Aug | 4 | 31-Aug | 22 | M | male |
| H19-4 | Hex | 4-Aug | 5-Aug | 4 | 10/11-Aug | 5 or 6 | 31-Aug | 21 | M | male |
| H33-1 | GFP | 28-Jul | 29-Jul | 3 | 29-Jul | 0 | 20-Aug | 22 | M | male |
| H33-2 | GFP | 28-Jul | 29-Jul | 3 | 29-Jul | 0 | 20-Aug | 22 | M | male |
| H33-3 | GFP | 28-Jul | 29-Jul | 4 | 30-Jul | 1 | 20-Aug | 21 | M | male |
| H33-4 | GFP | 28-Jul | 29-Jul | 4 | 31-Jul | 2 | 20-Aug | 20 | M | male |
| H33-5 | GFP | 28-Jul | 29-Jul | 4 | 4-Aug | 6 | 25-Aug | 21 | M | male |
| H33-6 | GFP | 28-Jul | 29-Jul | 4 | 1-Aug | 3 | 22-Aug | 21 | M | male |
| H33-7 | GFP | 4-Aug | 4-Aug | 2 | 5-Aug | 1 | 27-Aug | 21 | M | male |
| H33-8 | GFP | 4-Aug | 5-Aug | 4 | 7-Aug | 2 | 29-Aug | 22 | M | male |
| H33-9 | GFP | 4-Aug | 5-Aug | 4 | 8-Aug | 3 | 30-Aug | 22 | F | bioassay |
| H33-10 | GFP | 4-Aug | 5-Aug | 4 | 8-Aug | 3 | 29-Aug | 22 | M | male |
| H33-11 | GFP | 4-Aug | 5-Aug | 4 | 9-Aug | 4 | 31-Aug | 22 | F | bioassay |
| H33-12 | GFP | 4-Aug | 5-Aug | 4 | 11-Aug | 6 | 2-Sep | 22 | M | male |
| H35-1 | GFP | 29-Jul | 29-Jul | 2 | 30-Jul | 1 | 20-Aug | 21 | F | <4 feedings |
| H25-2 | GFP | 29-Jul | 30-Jul | 4 | 1-Aug | 2 | 22-Aug | 21 | F | bioassay |
| H35-3 | GFP | 29-Jul | 30-Jul | 4 | 2-Aug | 3 | 22-Aug | 20 | F | bioassay |
| H35-4 | GFP | 29-Jul | 30-Jul | 4 | 2-Aug | 3 | 22-Aug | 20 | F | bioassay |
| H35-5 | GFP | 29-Jul | 30-Jul | 4 | 2-Aug | 3 | 22-Aug | 20 | F | bioassay |
| H35-6 | GFP | 29-Jul | 30-Jul | 4 | 5-Aug | 6 |  |  |  | cannibalized |
| H35-7 | GFP | 4-Aug | 4-Aug | 2 | 5-Aug | 1 |  |  |  | cannibalized |
| H35-8 | GFP | 4-Aug | 5-Aug | 4 | 6-Aug | 1 |  |  |  | cannibalized |
| H35-9 | GFP | 4-Aug | 5-Aug | 4 | 8-Aug | 3 | 26-Aug | 18 | M | male |
| H35-10 | GFP | 4-Aug | 5-Aug | 4 | 8-Aug | 3 |  |  |  | cannibalized |
| H35-11 | GFP | 4-Aug | 5-Aug | 4 | 9-Aug | 4 |  |  |  | cannibalized |
| H35-12 | GFP | 4-Aug | 5-Aug | 4 | 11-Aug | 6 |  |  |  | cannibalized |
| L2-1 | GFP | 29-Jul | 29-Jul | 1 | 29-Jul | 0 |  |  |  | destroyed |
| L2-2 | GFP | 29-Jul | 29-Jul | 1 | 29-Jul | 0 |  |  |  | destroyed |
| L2-3 | GFP | 29-Jul | 30-Jul | 4 | 31-Jul | 1 |  |  |  | destroyed |
| L2-4 | GFP | 29-Jul | 30-Jul | 4 | 1-Aug | 2 |  |  |  | destroyed |
| L2-5 | GFP | 29-Jul | 30-Jul | 4 | 1-Aug | 2 |  |  |  | destroyed |
| L2-6 | GFP | 29-Jul | 30-Jul | 4 | 4-Aug | 5 |  |  |  | destroyed |
| L11-1 | Hex | 29-Jul | 30-Jul | 4 | 4-Aug | 5 | 25-Aug | 21 | M | male |
| L11-2 | Hex | 29-Jul | 30-Jul | 4 | 1-Aug | 2 | 21-Aug | 20 | M | male |
| L11-3 | Hex | 29-Jul | 30-Jul | 4 | 2-Aug | 3 | 23-Aug | 21 | M | male |
| L11-4 | Hex | 29-Jul | 30-Jul | 4 | 5-Aug | 6 | 26-Aug | 21 | M | male |
| L11-5 | Hex | 4-Aug | 5-Aug | 4 | 9-Aug | 4 | 31-Aug | 22 | M | male |
| L11-6 | Hex | 4-Aug | 5-Aug | 4 | 9-Aug | 4 | 31-Aug | 22 | M | male |
| L11-7 | Hex | 4-Aug | 5-Aug | 4 | 12-Aug | 7 | 3-Sep | 22 | M | male |
| L11-8 | Hex | 4-Aug | 5-Aug | 4 | 12-Aug | 7 |  |  |  | cannibalized |
| L13-1 | Hex | 2-Aug | 3-Aug | 4 | 7-Aug | 4 |  |  |  | destroyed |
| L13-2 | Hex | 2-Aug | 3-Aug | 4 | 8-Aug | 5 |  |  |  | destroyed |
| L13-3 | Hex | 2-Aug | 3-Aug | 4 | 4-Aug | 1 |  |  |  | destroyed |
| L13-4 | Hex | 2-Aug | 3-Aug | 4 | 5-Aug | 2 |  |  |  | destroyed |
| L15-1 | Hex | 2-Aug | 3-Aug | 4 | 5-Aug | 2 | 25-Aug | 20 | M | male |
| L15-2 | Hex | 2-Aug | 3-Aug | 4 | 6-Aug | 3 | 25-Aug | 19 | M | male |
| L15-3 | Hex | 2-Aug | 3-Aug | 4 | 7-Aug | 4 | 26-Aug | 18 | M | male |
| L15-4 | Hex | 2-Aug | 3-Aug | 4 | 7-Aug | 4 | 26-Aug | 18 | M | male |
| L15-5 | Hex | 2-Aug | 3-Aug | 4 | 9-Aug | 6 | 28-Aug | 20 | F | bioassay |
| L15-6 | Hex | 2-Aug | 3-Aug | 4 | 8-Aug | 5 | 28-Aug | 19 | M | male |
| L15-7 | Hex | 2-Aug | 3-Aug | 4 | 9-Aug | 6 | 31-Aug | 22 | M | male |
| L15-8 | Hex | 2-Aug | 3-Aug | 4 | 8-Aug | 5 | 28-Aug | 19 | M | male |
| L15-9 | Hex | 4-Aug | 4-Aug | 1 | 4-Aug | 0 | 24-Aug | 20 | F | <4 feedings |
| L15-10 | Hex | 4-Aug | 6-Aug | 4 | 7-Aug | 1 | 28-Aug | 22 | M | male |
| L15-11 | Hex | 4-Aug | 4-Aug | 2 |  |  |  |  |  | cannibalized |
| L15-12 | Hex | 4-Aug | 4-Aug | 2 |  |  |  |  |  | cannibalized |
| L15-13 | Hex | 5-Aug | 6-Aug | 4 | 8-Aug | 2 | 31-Aug | 23 | M | male |
| L15-14 | Hex | 5-Aug | 6-Aug | 4 | 10/11-Aug | 4 or 5 | 31-Aug | 21 | M | male |
| L15-15 | Hex | 5-Aug | 6-Aug | 4 | 11-Aug | 5 | 3-Sep | 23 | M | male |
| N26-1 | GFP | 8-Aug | 8-Aug | 1 |  |  |  |  |  | cannibalized |
| N26-2 | GFP | 8-Aug | 9-Aug | 4 | 12-Aug | 3 | 1-Sep | 20 | F | bioassay |
| N26-3 | GFP | 8-Aug | 9-Aug | 4 |  |  |  |  |  | never cocooned |
| N26-4 | GFP | 8-Aug | 9-Aug | 4 |  |  |  |  |  | never cocooned |
| N26-5 | GFP | 8-Aug | 9-Aug | 4 |  |  |  |  |  | never cocooned |
| N28-1 | Hex | 8-Aug | 8-Aug | 1 |  |  |  |  |  | cannibalized |
| N28-2 | Hex | 8-Aug | 10-Aug | 4 |  |  |  |  |  | never cocooned |
| N28-3 | Hex | 8-Aug | 8-Aug | 1 |  |  |  |  |  | cannibalized |
| N28-4 | Hex | 8-Aug | 9-Aug | 1 |  |  |  |  |  | cannibalized |
| N28-5 | Hex | 9-Aug | 10-Aug | 4 |  |  |  |  |  | cannibalized |
| N28-6 | Hex | 8-Aug | 9-Aug | 4 | 10-Aug | 1 | 31-Aug | 21 | M | male |
| N28-7 | Hex | 8-Aug | 9-Aug | 4 | 10-Aug | 1 |  |  |  | cannibalized |
| N28-8 | Hex | 8-Aug | 9-Aug | 4 |  |  |  |  |  | never cocooned |
| N28-9 | Hex | 8-Aug | 9-Aug | 4 | 18-Aug | 9 | 2-Sep | 15 | M | male |
| N28-10 | Hex | 8-Aug | 9-Aug | 4 |  |  |  |  |  | never cocooned |
