# Supplementary material for: Development of an RNA Interference Tool, Characterization of Its Target, and an Ecological Test of Caste Differentiation in the Eusocial Wasp Polistes
Source: PLoS One. 2011 Nov 1;6(11):e26641. doi: 10.1371/journal.pone.0026641 (PMC3206021; doi:10.1371/journal.pone.0026641)
Supplement: Table S3 — Specimen identities, dsRNA treatments, and bioassay response data for the 22 adult wasps used for all analyses. (DOC) [file pone.0026641.s006.doc]

| Specimen ID | Treatment | Ovary class | Wing length (mm) | Days to cocoon | Cocoon duration | Total days | Caterpillars eaten | Eggs laid |
| --- | --- | --- | --- | --- | --- | --- | --- | --- |
|
| A17-1 | GFP | 1.2 | 14.39 | 6 | 17 | 23 | 5 |  |
| H13-1 | GFP | 1.2 | 14.1 | 7 | 19 | 26 | 4 |  |
| H13-2 | GFP | 0 | 14.41 | 7 | 21 | 28 | 4 |  |
| H13-3 | GFP | 0 | 14.58 | 5 | 20 | 25 | 8 |  |
| H33-9 | GFP | 0.6 | 15.24 | 3 | 22 | 25 | 5 |  |
| H33-11 | GFP | 1.2 | 15.07 | 4 | 22 | 26 | 2 |  |
| H35-2 | GFP | 2.2 | 14.94 | 2 | 21 | 23 | 6 |  |
| H35-3 | GFP | 2.4 | 14.94 | 3 | 20 | 23 | 6 |  |
| H35-4 | GFP | 2 | 14.96 | 3 | 20 | 23 | 6 |  |
| H35-5 | GFP | 1 | 14.89 | 3 | 20 | 23 | 4 |  |
| N26-2 | GFP | 3 | 13.36 | 3 | 20 | 23 | 6 | 1 |
| A16-2 | HEX | 0.6 | 14.37 | 7 | 20 | 27 | 6 |  |
| A16-3 | HEX | 0.4 | 15.56 | 7 | 19 | 26 | 6 |  |
| A16-4 | HEX | 1.6 | 16 | 7 | 19 | 26 | 4 |  |
| A16-5 | HEX | 0.8 | 15.44 | 7 | 19 | 26 | 5 |  |
| H1-3 | HEX | 0.8 | 14.84 | 8 | 21 | 29 | 4 |  |
| H1-4 | HEX | 1 | 15.34 | 7 | 19 | 26 | 8 |  |
| H1-8 | HEX | 1 | 14.41 | 6 | 19 | 25 | 3 |  |
| H1-12 | HEX | 1.6 | 14.79 | 9 | 18 | 27 | 6 |  |
| H16-1 | HEX | 1.4 | 14.98 | 4 | 21 | 25 | 4 |  |
| H16-5 | HEX | 0.6 | 15.26 | 2 | 21 | 23 | 4 |  |
| L15-5 | HEX | 0.2 | 14.09 | 6 | 20 | 26 | 6 |  |
